# Involvement of the Same TNFR1 Residue in Mendelian and Multifactorial Inflammatory Disorders

**DOI:** 10.1371/journal.pone.0069757

**Published:** 2013-07-24

**Authors:** Isabelle Jéru, Serge Charmion, Emmanuelle Cochet, Bruno Copin, Philippe Duquesnoy, Maria Teresa Mitjavila Garcia, Gaëlle Le Borgne, Pascal Cathebras, Jacques Gaillat, Sonia Karabina, Catherine Dodé, Peter Lohse, Véronique Hentgen, Serge Amselem

**Affiliations:** 1 UMR_S933, Institut National de la Santé et de la Recherche Médicale (INSERM), Paris, France; 2 UMR_S933, Université Pierre et Marie Curie-Paris6 (UPMC), Paris, France; 3 Service de Génétique et d’Embryologie médicales, Hôpital Trousseau, Assistance Publique-Hôpitaux de Paris (AP-HP), Paris, France; 4 Département hospitalo-universitaire Inflammation – Immunopathology – Biotherapy, UPMC, AP-HP, INSERM and Centre National de la Recherche Scientifique (CNRS), Paris, France; 5 Service de Médecine Interne, Centre Hospitalier Universitaire de Saint-Etienne, Saint-Etienne, France; 6 UMR_S935, INSERM, Villejuif, France; 7 Service des Maladies Infectieuses, Centre Hospitalier de la Région d'Annecy, Pringy, France; 8 Service de Biochimie et Génétique Moléculaire, Hôpital Cochin, AP-HP, Paris, France; 9 Department of Clinical Chemistry - Großhadern, University of Munich, Munich, Germany; 10 Institute of Laboratory Medicine and Human Genetics, Singen, Germany; 11 French Reference Centre for Auto-Inflammatory Diseases (CeRéMAI), Centre Hospitalier de Versailles, Le Chesnay, France; INSERM, France

## Abstract

**Objectives:**

*TNFRSF1A* is involved in an autosomal dominant autoinflammatory disorder called *TNFR*-associated periodic syndrome (TRAPS). Most *TNFRSF1A* mutations are missense changes and, apart from those affecting conserved cysteines, their deleterious effect remains often questionable. This is especially true for the frequent R92Q mutation, which might not be responsible for TRAPS per se but represents a susceptibility factor to multifactorial inflammatory disorders. This study investigates TRAPS pathophysiology in a family exceptional by its size (13 members) and compares the consequences of several mutations affecting arginine 92.

**Methods:**

*TNFRSF1A* screening was performed by PCR-sequencing. Comparison of the 3-dimensional structure and electrostatic properties of wild-type and mutated TNFR1 proteins was performed by *in silico* homology modeling. TNFR1 expression was assessed by FACS analysis, western blotting and ELISA in lysates and supernatants of HEK293T cells transiently expressing wild-type and mutated TNFR1.

**Results:**

A *TNFRSF1A* heterozygous missense mutation, R92W (c.361C>T), was shown to perfectly segregate with typical TRAPS manifestations within the family investigated (p<5.10^−4^). It was associated with very high disease penetrance (0.9). Prediction of its impact on the protein structure revealed local conformational changes and alterations of the receptor electrostatic properties. R92W also impairs the TNFR1 expression at the cell surface and the levels of soluble receptor. Similar results were obtained with R92P, another mutation previously identified in a very small familial form with incomplete penetrance and variable expressivity. In contrast, TNFR1-R92Q behaves like the wild-type receptor.

**Conclusions:**

These data demonstrate the pathogenicity of a mutation affecting arginine 92, a residue whose involvement in inflammatory disorders is deeply debated. Combined with previous reports on arginine 92 mutations, this study discloses an unusual situation in which different amino acid substitutions at the same position in the protein are associated with a clinical spectrum bridging Mendelian to multifactorial conditions.

## Introduction

Hereditary recurrent fevers (HRF) are part of the emerging group of autoinflammatory disorders, which result from abnormal regulation of the innate immune system. Among them, the autosomal dominant tumor necrosis factor receptor (TNFR)-associated periodic syndrome (TRAPS) has been associated with mutations in *TNFRSF1A* [1], a widely expressed gene encoding the 55 kDa TNF receptor (also known as TNFR1 or p55). TNFR1 mediates TNF signalling and induces either pro-inflammatory pathways or apoptosis. The receptor comprises 4 extracellular cysteine-rich domains (CRD1–4), a transmembrane region, and an intracellular death domain (DD). The clinical diagnosis of TRAPS is difficult to establish due to a lack of objective diagnostic criteria. Patients present with recurrent episodes of fever, systemic inflammation, and various seemingly-unprovoked inflammatory signs. Attacks usually last from a few days to several weeks. The most frequent manifestations observed in TRAPS include fever, myalgia with underlying fasciitis, migratory skin rashes, and abdominal pain. The inflammatory attacks can also be associated with pericarditis, arthralgia, arthritis, and ocular manifestations (conjunctivitis, periorbital edema). Like in other autoinflammatory disorders, sustained elevation of inflammatory markers can lead to AA amyloidosis [2].

Numerous sequence variations, corresponding mostly to missense mutations have been identified in the *TNFRSF1A* gene (Infevers database, http://fmf.igh.cnrs.fr/ISSAID/infevers/). Many of them involve highly conserved cysteine residues, which are important for disulfide bond formation, and TNFR1 proper folding. These mutations are usually found in familial forms of TRAPS and associated with high disease penetrance [3]–[4]. They are generally associated with a severe phenotype and increased prevalence of renal amyloidosis [3]. In contrast, the pathogenicity of other sequence variations remains a subject of debate. This is especially true for the frequently identified R92Q variant whose deleterious effect in TRAPS is far from clear. Indeed, this sequence variation is usually found in sporadic cases. Family studies revealed that R92Q is associated with a low disease penetrance [3]–[8] and, in some cases, it does not even segregate with the disease phenotype [5]–[6]. Consistently, this variant is associated with milder clinical forms and higher rate of spontaneous resolution [2]–[4], [6]–[7]. Its frequency in the general population has been reported to reach up to 5% [3], [7], [9]–[12]. A number of studies also reported a role for R92Q in the susceptibility to multifactorial inflammatory conditions such as early arthritis [3], AA amyloidosis in juvenile idiopathic arthritis [13], thrombotic complications in Behçet’s disease [14], idiopathic recurrent pericarditis [15], myocardial infarction [9], and multiple sclerosis [16]–[17]. Altogether, these data support the notion that R92Q is not a mutation responsible for TRAPS per se, but rather represents a sequence variation with mild pro-inflammatory effects (e.g. [3], [5], [18]). Notably, another mutation affecting arginine 92 (R92P) has been identified in a Dutch family of small size with HRF symptoms. In that case, the disease penetrance was low since, among 5 carriers, 1 presented atypical isolated irritable bowel disease, 2 were unaffected [19], and only 2 had symptoms evocative of TRAPS. This observation further raised the question of the clinical impact of sequence variations involving arginine 92.

In the current study, screening of *TNFRSF1A* in a very large family with TRAPS revealed a third mutation involving the same residue, R92W. This led us to compare the impact of this alteration with that of other sequence variations affecting arginine 92.

## Patients and Methods

### Patients and Ethics Statement

This study was approved by the Comité de Protection des Personnes Ile-de-France 5, Paris, France. This study investigates a family referred to one of the French national centers for molecular diagnosis of HRF. Informed written consent for genetic studies was given by all participants. Genotyping was performed only in adults. Clinical features were recorded on a standardized form.

### Molecular Analysis

gDNA was extracted from peripheral blood leukocytes using a commercial kit (FlexiGene, Qiagen). Exons and flanking intronic sequences of *TNFRSF1A* were amplified by PCR, purified, sequenced with the Big Dye Terminator sequencing kit (Applied Biosystems), and run on an ABI 3730×l automated sequencer. Sequences were analyzed with SeqScape software (Applied Biosystems). The ten exons of the gene were screened in one family member. In relatives, the presence of the R92W mutation was searched for and confirmed by forward and reverse sequencing.

### Prediction of TNFR1 3-dimensional (3D) Structure and Electrostatic Properties

Models of wild-type and mutated TNFR1 3D structures were generated using the receptor monomer A chain from the Protein Data Bank crystal structure 1EXT as template. Homology modeling was performed using Modeller software v.9.8 [20]. Accuracy of output structures from Modeller were further assessed using Procheck v.3.4.3 [21]. Graphic displays were generated using the PyMOL Molecular Graphic System v0.99. Electrostatic properties of the models were predicted with the vacuum electrostatics PyMOL plug-in.

### Plasmid Constructs

*TNFRSF1A* cDNA was amplified by RT-PCR using total RNA from leukocytes as template. The TNFR1 expression vector (designated pTNFR1-WT) was obtained by cloning the wild-type *TNFRSF1A* cDNA into the pcDNA3.1 expression plasmid. Site-directed mutagenesis (QuickChange, Stratagene) was performed according to the manufacturer’s instructions to generate expression plasmids carrying different mutations: pTNFR1-P46L, pTNFR1-T50M, pTNFR1-R92Q, pTNFR1-R92P, pTNFR1-R92W, pTNFR1-C70S, and pTNFR1-C73R. All constructs were checked by sequencing.

### Enzyme-linked Immunosorbent Assay (ELISA)

HEK293T cells (ATCC) were cultured in 6-well plates (5.10^5^/well) in DMEM, penicillin (100 IU.mL^−1^) and streptomycin (100 µg.mL^−1^). Cells were transfected (FuGENE®, Promega) with 500 ng of the WT and mutated forms of the TNFR1 expression plasmids according to the manufacturer’s protocol. After transfection, cells were cultured 24 h in serum-free medium. Cell supernatants were then collected and centrifuged at 300 g for 10 minutes to remove cellular debris. ELISA assays were performed on cell supernatants to detect soluble forms of TNFR1 by means of a commercial kit (DuoSet ELISA human sTNFR1/TNFRSF1A, R&D Systems), according to the manufacturer’s instructions.

### Western Blotting

The same cells transiently expressing TNFR1 were used as those prepared for ELISA. 24 h after transfection, cell supernatants were centrifuged at 300 g for 10 minutes to remove cellular debris and concentrated 16-fold by using Amicon Ultra-4 Centrifugal Filter units (Millipore). Cells were also harvested, and lysed in a buffer containing 20 mM sodium phosphate pH 7.5, 0.1% SDS (w/v), 50 mM β-mercaptoethanol and 0.75% NP-40 (w/v). 40 µg of proteins were then subjected to sodium dodecyl sulfate-polyacrylamide gel electrophoresis and transferred onto PVDF membranes. Western blotting was performed with a goat polyclonal anti-TNFR1 antibody (AF225, R&D Systems), a mouse monoclonal anti-α tubulin antibody (Santa-Cruz Biotechnology), as well as HRP-conjugated anti-goat R&D Systems) and anti-mouse (Sigma-Aldrich) antibodies. Detection was performed with chemiluminescence reagents (Pierce) and ChemiDoc XRS detection system (Biorad).

### Fluorescence-activated Cell Sorter (FACS) Analysis

HEK293T cells were cultured in 6-well plates, as described previously, and transfected with 1 µg of the TNFR1 expression plasmids. 24 h after transfection, cells were washed, resuspended in PBS containing 1% bovine serum albumin and incubated 30 minutes on ice with either a mouse monoclonal phycoerythrin (PE)-conjugated anti-TNFR1 antibody (FAB225P, R&D Systems) or with a goat polyclonal anti-TNFR1 antibody (AF225, R&D Systems). In the latter case, cells were washed and incubated with a secondary anti-goat antibody conjugated with Alexa Fluor 488 (Life Technologies). Background staining was evaluated using a mouse isotype-matched control antibody (PE-conjugated IgG1, R&D Systems) in the first situation, and a normal goat IgG control antibody (R&D Systems) followed by the same Alexa fluor-conjugated secondary antibody in the second situation. After cell washing, fluorescence was acquired and analysed with a FACSCalibur flow cytometer (Becton Dickinson), using the CellQuest Pro Analysis software (Becton Dickinson). Dead cells were excluded using 7-aminoactinomycine D (Sigma-Aldrich). To perform intracellular staining, cells were fixed and permeabilized with the Fix and Perm kit (Invitrogen), according to the manufacturer’s instructions.

## Results

### Clinical Presentation

**Figure 1:**
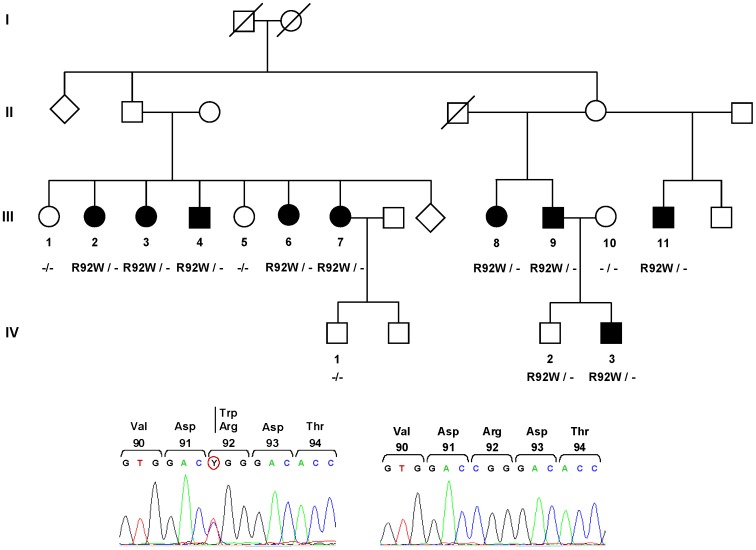
Genealogical tree and mutation analysis. Filled symbols represent patients with TRAPS; open squares and circles indicate healthy individuals. No clinical information was available for individuals from generations I and II. A sequencing chromatogram showing the mutation identified in family members III.2, III.3, III.4, III.6, III.7, III.8, III.9, III.11, IV.2, and IV.3 is presented below. The sequence in healthy relatives (III.1, III.5, III.10, IV.1) is shown as a control. The heterozygous transition generating the missense mutation is encircled.

The large French family investigated herein comprised 9 affected individuals, 4 asymptomatic members, and one healthy spouse (Figure 1). We had no clinical information regarding individuals from generations I and II. All patients presented with symptoms consistent with a diagnosis of TRAPS (Table 1). The age at onset of symptoms in this family was between 11 and 41 years. Depending on the patients, the frequency of inflammatory episodes varied from 1 per month to less than 1 per year (Table 1). Triggering factors were observed only in two patients: psychological stress was reported by one individual, season change and physical exertion by a second family member. All patients reported recurrent fever attacks, lasting 8 to 30 days, which resolved spontaneously. Fever was high in 7 affected family members (at least 39°C) and moderate in two patients (38–38.5°C). Abdominal pain was present in all patients and was associated with diarrhea or constipation in five of them. Myalgia and arthralgia were observed in half of the affected members. Two patients also presented thoracic pain and/or pericarditis. Headaches, buccal aphtosis and asthenia were each reported in one family member. CRP levels were found to be elevated during attacks in all patients tested (67–300 mg.L^−1^), consistent with an autoinflammatory disorder. One patient had moderate proteinuria (100 mg/24 h). Treatment of attacks with corticoids (prednisone, ∼ 0.5 mg/kg/day) was tested in two family members, leading to rapid clinical remission and normalization of biological parameters.

**Table 1:** Clinical presentation of the patients carrying the R92W mutation.

	III.2	III.3	III.4	III.6	III.7	III.8	III.9	III.11	IV.3
Age at onset of attacks (years)	27	20	36	37	20	20	12	41	11
Frequency of attacks	1–2/year	<1/year	<1/year	<1/month	<1/month	<1/month	1/year	<1/year	1–2/year
Attack duration (days)	8–21	30	20–30	15–21	21	15–20	nd*	15–20	10–30
Triggering factor	no	no	psychological stress	no	no	no	no	no	season (spring, autumn),physical stress
Fever	yes (38.5°C)	yes (39°C)	yes (38–39°C)	yes (39°C)	yes (38–39°C)	yes (38.5°C)	yes (39°C)	yes (39°C)	yes (39°C)
Abdominalmanifestations	pain, diarrhea	pain	pain, diarrhea	pain, diarrhea,constipation	pain, constipation	pain, diarrhea, constipation	pain	pain	pain
Articular signs	no	arthralgia	no	no	arthralgia	arthralgia	no	no	arthralgia
Myalgia	no	yes	yes	no	yes	no	no	no	yes
Thoracic signs	no	pericarditis (3 times)	thoracic pain	no	no	no	no	no	no
Cutaneous manifestations	no	no	no	no	no	no	no	no	no
Lymphatic signs	no	no	no	no	no	no	no	no	no
Headaches	no	no	no	no	no	no	no	yes	no
Hearing impairment	no	no	no	no	no	no	no	no	no
Ocular manifestations	no	no	no	no	no	no	no	no	no
Neurologicmanifestations	no	no	no	no	no	no	no	no	no
Proteinuria (mg.24 h^−1^)	no	no	no	no	no	no	no	no	100
CRP levels (mg.L^−1^)	107	240	67	143	300	nd	nd	69	168
Other signs	buccal aphtosis	no	no	no	no	no	no	asthenia	no

* nd: not determined.

### Identification of a *TNFRSF1A* Missense Mutation

A heterozygous sequence variation (c.361C>T), designated R92W (according to the commonly used nomenclature [1]) was identified in *TNFRSF1A* exon 4 (Figure 1). This mutation is also called p.Arg121Trp based on the reference sequence NM_001065.3 and according to the official nomenclature (http://www.hgvs.org/) which takes into account the 29 amino-acid signal peptide. Notably, 9 out of the 10 family members carrying this mutation presented a typical form of TRAPS. The only asymptomatic individual (IV.2) is still relatively young (30 years old) as compared to three of his affected relatives who had their first inflammatory attacks at the age of 36, 37 and 41 respectively, so that we cannot exclude that he might develop manifestations later in life. Three healthy adult relatives (III.1, III.5 and IV.1) who agreed to participate in the study did not carry the mutation. Altogether, these results clearly show that the mutation segregates with the disease phenotype within the family. Indeed, considering the number of members investigated here (n = 13), the risk to observe such a segregation by coincidence is lower than 5.10^−4^. Screening of the ten *TNFRSF1A* exons and flanking intronic sequences in one affected family member did not reveal any additional mutation. The R92W sequence variation was not found in population-matched DNA controls originating from France (n = 106), indicating that this variant is not a frequent polymorphism. Finally, alignment of TNFR1 protein sequences from different species showed that the arginine 92 residue is well conserved throughout evolution.

### Consequences of Mutations Affecting Arginine 92 on TNFR1 3-D Structure and Electrostatic Properties

**Figure 2:**
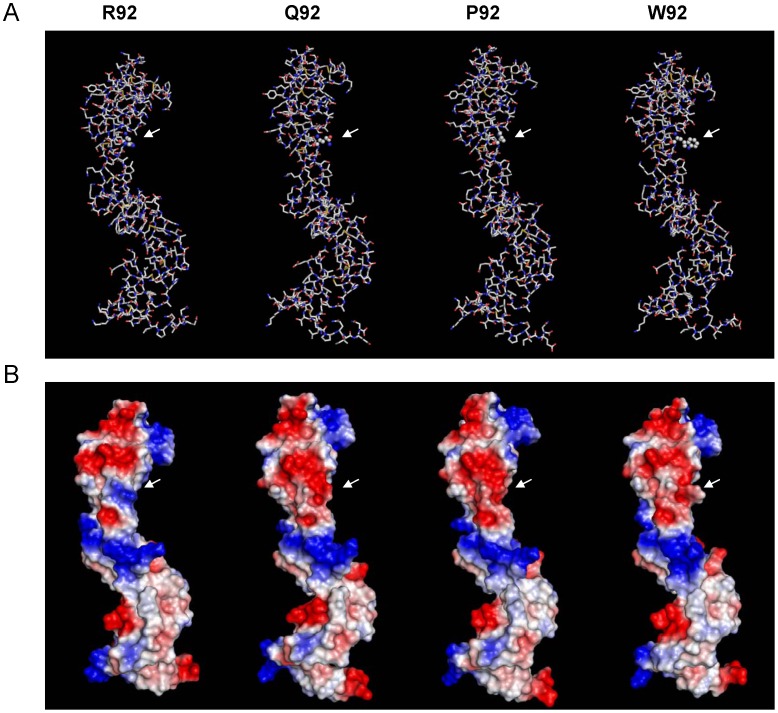
Modeling of structural and electrostatic changes induced by mutations affecting arginine 92 on the TNFR1 ectodomain. **A,** 3D structures of wild-type and mutated TNFR1 proteins. Protein models were generated by homology modeling. Carbon, oxygen, nitrogen and sulphur atoms are shown in white, red, blue and yellow, respectively. Residue 92 is represented with balls and other amino acids with sticks. **B,** Electrostatic properties of wild-type and mutated TNFR1 proteins. Electrostatic potentials are mapped on the surface of the TNFR1 3D structure. Blue color indicates regions of positive potential, whereas red depicts negative potential values. The position of the residue at position 92 is indicated by a white arrow.

The R92W mutation is located within the β turn of loop 3 of the CRD2 domain of the receptor and introduces a polarity change. We evaluated by *in silico* homology modeling its structural impact, as well as that of other variations affecting the same residue (R92P, R92Q). As compared to wild-type TNFR1, prediction of the 3D structure of the proteins carrying the R92W, R92P, and R92Q mutations disclosed conformational changes in the vicinity of the mutated residue with moderate consequences for the overall protein structure (Figure 2A). In addition, electrostatic modeling revealed that the R92W, R92P, and R92Q mutations strongly affect polarity in the region surrounding residue 92, as compared to the wild-type receptor (Figure 2B). Mild differences were also seen in more distant parts of the receptor ectodomain.

### Effects of Mutations Affecting Arginine 92 on TNFR1 Expression and Trafficking

**Figure 3:**
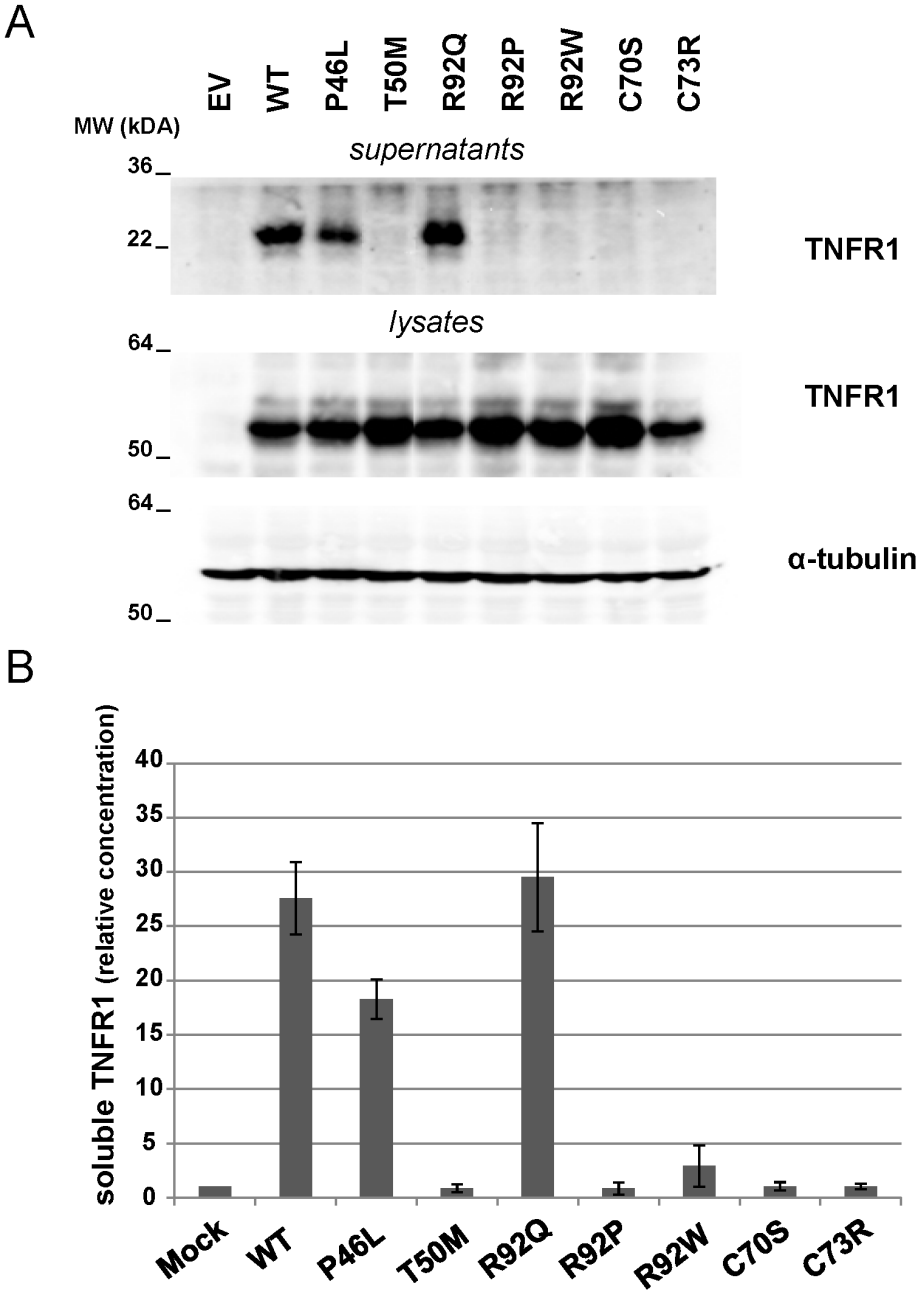
Comparison of sTNFR1 levels for the WT and mutated forms of the receptor. **A**, Detection of TNFR1 by western blotting in supernatants and lysates from HEK293T cells transiently expressing the WT and mutated forms of the receptor. The data presented are representative of those obtained in three independent experiments. **B**, Measurement of soluble TNFR1 (sTNFR1) in supernatants from the same cells. Results from ELISA are presented as means ± SD of three independent experiments performed in duplicate. Data were normalized to the concentration measured in the mock condition.

**Figure 4:**
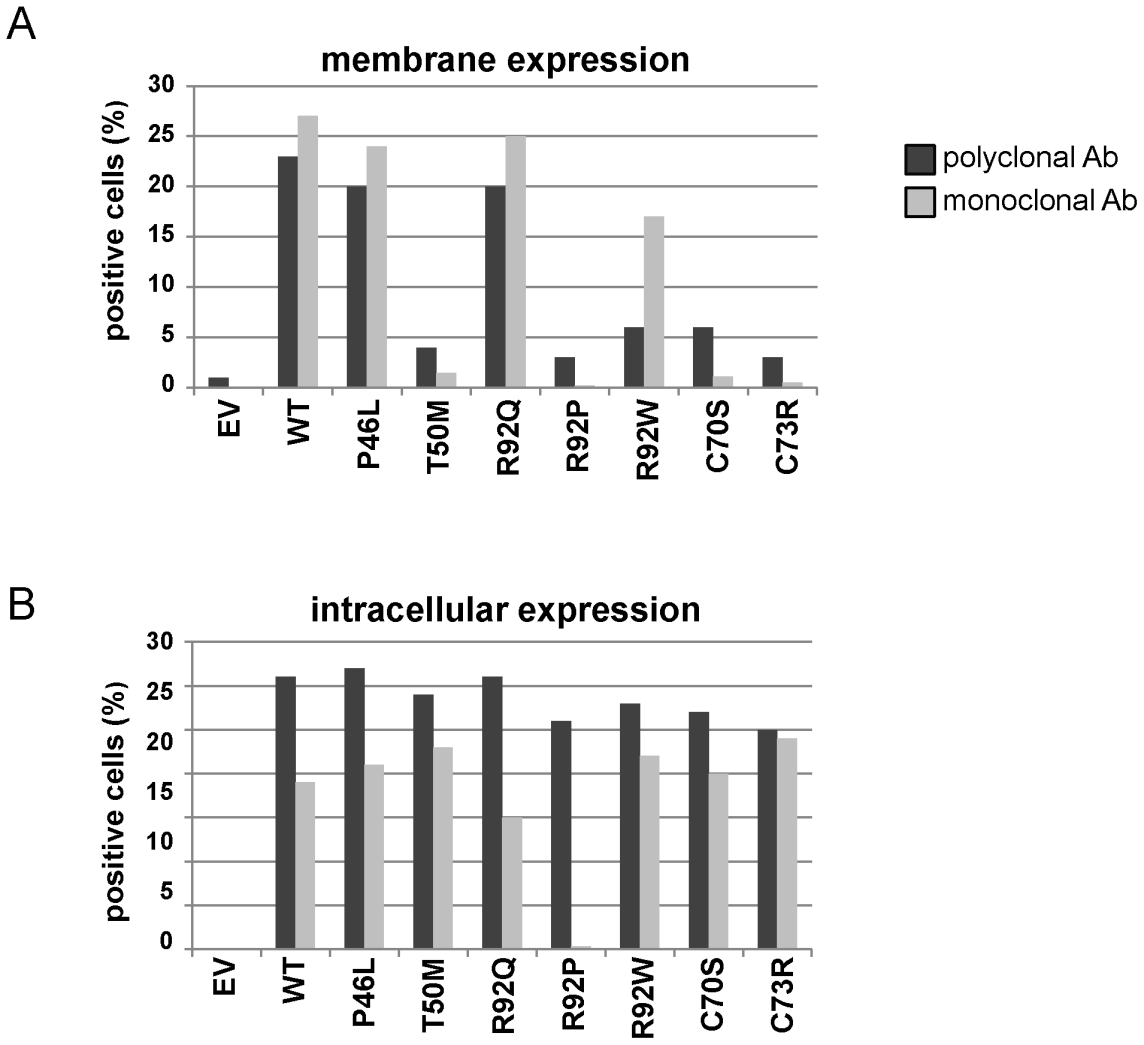
Trafficking of WT and mutated forms of TNFR1 at the cell surface. FACS analysis was performed in HEK293T cells transiently expressing the WT and mutated forms of the receptor. Cells were incubated either with a PE-conjugated monoclonal anti-TNFR1 antibody or with a goat polyclonal anti-TNFR1 antibody followed by an Alexa fluor-conjugated anti-goat antibody. **A,** Percentage of cells expressing TNFR1 at their surface. **B,** Percentage of cells expressing TNFR1 after permeabilization.

We then assessed the effect of mutations affecting arginine 92 on TNFR1 expression. To this purpose, we transfected plasmids encoding WT and mutated forms of the receptor into HEK293T cells. As controls, we generated expression vectors for several *TNFRSF1A* unambiguous mutations (T50M, C70S, C73R) as well as for P46L, which is considered as a polymorphism or as a mutation with very mild pro-inflammatory effects [6]. As shown by western blotting in cell lysates (Figure 3A), TNFR1 expression was similar for TNFR1-WT and all mutated forms of the receptor. Nevertheless, western blot and ELISA assays performed on cell supernatants revealed that the soluble form of the receptor (sTNFR1) was detectable only for cells expressing TNFR1-WT, TNFR1-P46L and TNFR1-R92Q (Figure 3A and 3B). These data demonstrate that R92W and R92P result in a dramatic diminution of the pool of sTNFR1, like unambiguous mutations. In contrast, R92Q behaves like the WT protein. We also evaluated the membrane expression of TNFR1 by FACS analysis. We first used a PE-conjugated monoclonal anti-TNFR1 antibody (R&D Systems) used previously by other teams [7], [22]–[23]. Consistent with a previous observation [22], the receptor carrying the R92P mutation was not recognized by this antibody (Figure 4), so that we could not conclude on its subcellular localization. The subsequent use of a polyclonal antibody (R&D Systems) showed that TNFR1-R92P is indeed expressed in the cytoplasm, whereas very low levels are detectable at the cell surface. As shown in Figure 4A, the percentage of cells expressing the receptor at their surface is higher in the presence of TNFR1-WT, TNFR1-P46L and TNFR1-R92Q than in the presence of TNFR1-T50M, TNFR1-C70S, TNFR1-C73R, TNFR1-R92P and TNFR1-R92W. The same experiment performed after cell permeabilization did not reveal a different intracellular expression of the different forms of the receptor (Figure 4B).

## Discussion

Most *TNFRSF1A* mutations identified to date correspond to missense changes and, apart from those affecting the conserved cysteine residues, their deleterious effect remains often questionable. Indeed, appropriate functional assays are difficult to set up on a routine basis and the great majority of patients currently referred for genetic testing correspond to sporadic cases. The current study clearly establishes the causative role in TRAPS of a mutation affecting arginine 92, a residue whose involvement in inflammatory disorders is deeply debated. This led us to discuss the wide clinical spectrum associated with different mutations of this TNFR1 residue.

One straightforward way to provide evidence for the pathogenicity of newly identified sequence variations is to investigate large families, which are however extremely rare. In the present study, we report a familial form of TRAPS, exceptional by its size, since it comprises 13 family members. The deleterious effect of the R92W mutation identified in patients is clearly demonstrated by its segregation with disease manifestations within the family (p<5.10^−4^). The fact that the R92W mutation was not found in matched-population controls also argues for its pathogenicity.

From a clinical viewpoint, all affected family members present a very typical form of TRAPS. All of them display long-lasting episodes associating fever, systemic inflammation and abdominal pain; none of them experience skin rash or ocular manifestations, which are also observed in some patients with TRAPS [2], [6]. Notably, though its deleterious effect could not be established at that time, the R92W mutation was previously identified in two sporadic cases (P. Lohse et al., personal communication in the Infevers database; Ravet et al. [6]). We contacted these two teams to determine if R92W was also associated with typical TRAPS manifestations in these cases. The patient reported in [6] is a man who presented less than one inflammatory episode per month characterized by high fever (40°C) and peritonitis. He experienced his first attacks at the age of 28. It was, however, not possible to get a more precise description of the disease phenotype a posteriori. The patient reported by P. Lohse et al. is a lady who presented with a severe form of the disease. Reassessment of her medical history revealed that she started her manifestations at the age of 14 with 3 to 4 inflammatory episodes per year lasting up to 14 days. Her symptoms included high fever (up to 40°C), myalgia, arthragia, and acute abdominal pain. Attacks were also marked by systemic inflammation (CRP levels: 360 mg.L^−1^) and hyperleukocytosis. She was treated by high-doses of steroids. She also had an appendicectomy, a tonsillectomy and a cholecystectomy because of cholecystolithiasis. At the age of 48, she developed urticaria and acute monoarthritis. Etanercept was then introduced (2 subcutaneous injections/week) leading to almost complete remission. By contrast, the R92Q variation is found in patients with heterogeneous and milder clinical presentations of TRAPS [2]–[4], [6]–[7], in individuals presenting with other multifactorial inflammatory disorders [3], [9], [13]–[17], as well as in the general population [3], [7], [9]–[12]. As for R92P, which was identified in a single family, it is associated with an intermediate disease phenotype characterized by variable expressivity with clinical signs ranging from isolated abdominal pain to TRAPS manifestations. Another striking point is the very high penetrance of the disease phenotype associated with the R92W mutation (0.9), in comparison with the low penetrance reported with R92P [19] or with R92Q [3]–[7].

Many data have been generated on the mechanisms underlying TRAPS, though its pathogenesis is not completely understood. It was proposed that TRAPS was due to impaired metalloprotease-dependent cleavage of membrane TNFR1 that produces soluble receptors [1]. Then, other reports performed in neutrophils and fibroblasts suggested that TRAPS might be due to defective TNF-induced apoptosis [7], [24]. It is now recognized that TRAPS-associated unambiguous mutations profoundly alter receptor trafficking, leading to retention of mutant receptors in the endoplasmic reticulum [22]–[23], [25], [26]–[27]. The subsequent ligand-independent aggregation would lead to defect in autophagy [23], aberrant signalling [28]–[29], and production of reactive oxygen species (ROS) [30]. This intracellular accumulation would result from conformational changes in the 3-D structure of the receptor [22]. In this context, we indeed showed by *in silico* homology modeling that R92W, R92P, and R92Q affect the protein electrostatic properties and, to a lesser extent, the TNFR1 3D structure. Nevertheless, it seems very difficult to predict the severity of the different mutations affecting arginine 92 when using only *in silico* models. FACS analysis performed with a polyclonal antibody revealed a decrease in the cell surface expression of the TNFR1 receptor carrying the R92P and R92W mutations. Supporting this observation, very low levels of sTNFR1 were observed in the supernatants of cells transiently expressing TNFR1-R92W, when only traces were detected in the presence of R92P and other *TNFRSF1A* unambiguous mutations (T50M, C70S, C73R). In contrast, great amounts of sTNFR1 were present in supernatants of cells transiently expressing TNFR1-R92Q which behaves like the wild-type protein. This latter result is consistent with previous reports that showed similar biological properties for the R92Q mutated receptor and the WT protein [1], [3], [7], [22]–[24], [26], [30].

**Figure 5:**
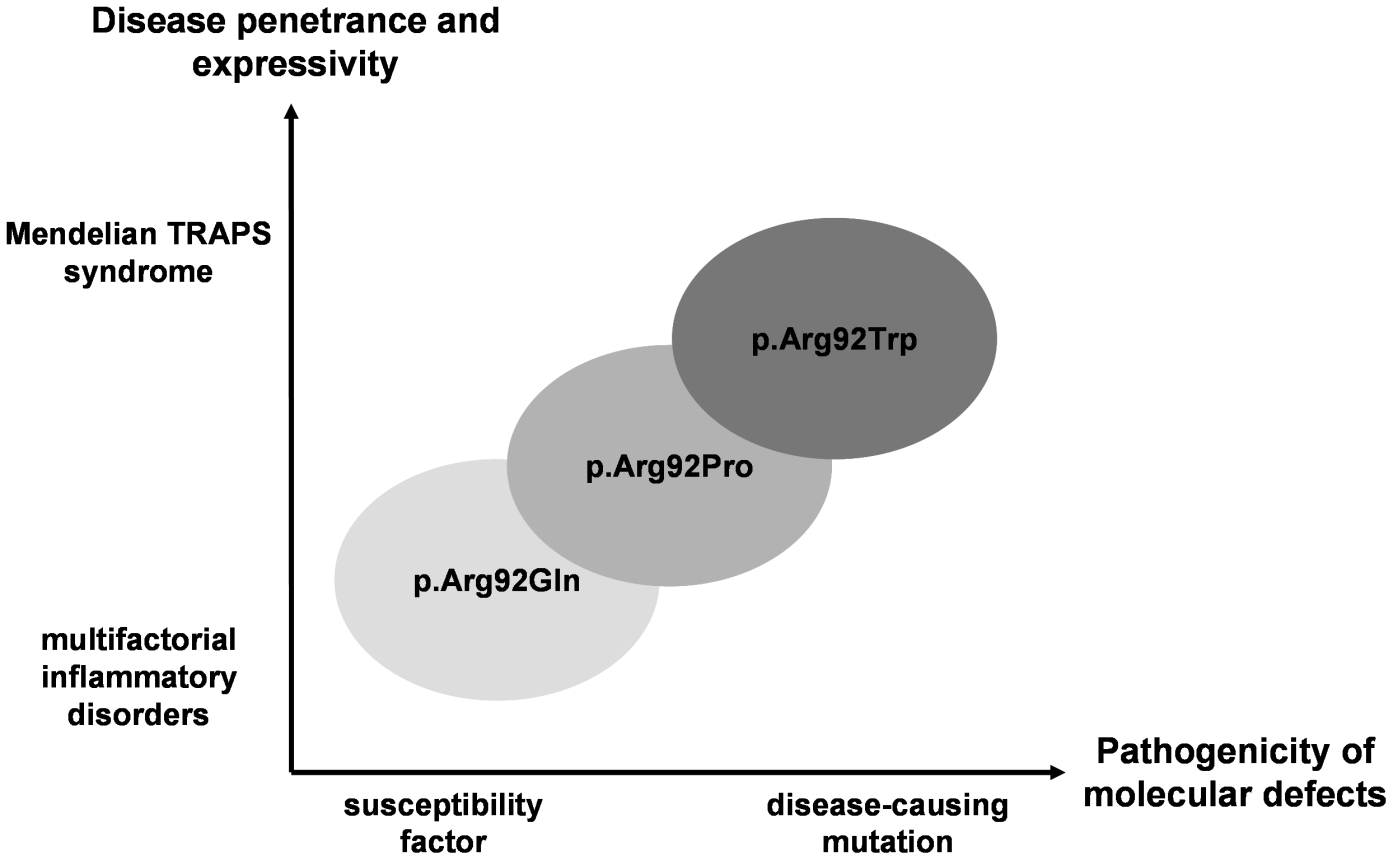
Schematic representation of the consequences of TNFR1 mutations affecting arginine 92 in inflammatory disorders. The pathogenicity associated with each mutation at residue 92 defines a continuum linking Mendelian to multifactorial inflammatory disorders.

Altogether, these data make clear that the R92W mutation is responsible for TRAPS. This will be of help to the diagnosis of patients carrying this mutation, since TRAPS still lacks objective diagnostic clinical criteria. This is all the more important as distinct therapeutic strategies are available for each HRF. This work allows further thought on a more general viewpoint. Several genes are known to be involved both in Mendelian and in multifactorial disorders (e.g. [31]–[33]), some of them playing a role in autoinflammatory diseases (e.g. [34]–[35]). Combined with previous clinical reports, the current data provide an example of a rare situation in which different amino acid substitutions at the same position in the protein are associated with a clinical spectrum bridging multifactorial to Mendelian disorders. The numerous R92Q-related inflammatory conditions (myocardial infarction, idiopathic recurrent pericarditis, early arthritis, AA amyloidosis in juvenile idiopathic arthritis, thrombosis in Behçet’s disease, multiple sclerosis) stand at one end of the spectrum, when the rare R92W-associated TRAPS syndrome appears at the other one. As for the R92P-associated disorder, it would lie in the middle of the clinical continuum, though only one small familial form has been reported to date (Figure 5). This direct link drawn between common inflammatory disorders and TRAPS, whose molecular aetiology, cellular bases, clinical manifestations and treatment have been the subject of intense investigations, could improve our understanding and management of complex traits, at least in some subsets of patients.
